# Estrogen receptor α activation modulates the gut microbiome and type 2 diabetes risk factors

**DOI:** 10.14814/phy2.15344

**Published:** 2022-06-13

**Authors:** Janelle A. Shiffler, Krista A. Goerger, Brittany K. Gorres‐Martens

**Affiliations:** ^1^ Exercise and Sport Sciences Department Augustana University Sioux Falls South Dakota USA; ^2^ 7352 Biology Department University of Sioux Falls Sioux Falls South Dakota USA

**Keywords:** estradiol, exercise, gut microbiome, type 2 diabetes

## Abstract

Estradiol and exercise can decrease risk factors associated with type 2 diabetes (T2D) including total body weight gain and abdominal fat gain. Estradiol functions through estrogen receptor (ER) α and ERβ. Some studies suggest that activation of ERα may provide protection against T2D. Female Wistar rats were ovariectomized and fed a high‐fat diet for 10 weeks and divided into the following 5 experimental groups: (1) no treatment (control), (2) exercise, (3) estradiol, (4) propylpyrazoletriyl (a selective ERα agonist), and (5) diarylpropionitrile (a selective ERβ agonist). ERα activation decreased the abundance of *Firmicutes*, and ERα and ERβ activation increased the abundance of *Bacteroidetes*. ERα activation decreased food consumption, and ERα and ERβ activation increased voluntary activity. Exercise was the only treatment to decrease the blood glucose and serum insulin levels. ERα activation, but not ERβ, increased hepatic protein expression of ACC and FAS and decreased hepatic protein expression of LPL. ERα activation also decreased hepatic mRNA expression of PPARα and PPARγ. This study elucidates the functions of estradiol by assessing specific activation of ERα and ERβ. As obesity increases the abundance of *Firmicutes* and decreases the abundance of *Bacteroidetes*, our study shows that ERα activation can restore the gut microbiome to non‐obese abundances. This study further provides novel insights into ERα’s role in hepatic fat metabolism via regulation of ACC, FAS, LPL, PPARα, and PPARγ.

## Graphical abstract

Estrogen receptor (ER) α activation restored the gut microbiome to non‐obese abundances. Exercise, but not ER activation, decreased the blood glucose and serum insulin levels. ERα activation increased hepatic protein expression of ACC and FAS, decreased hepatic protein expression of LPL, and decreased hepatic mRNA expression of PPARα and PPARγ.

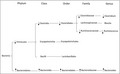

## 1 INTRODUCTION

In 2017, the International Diabetes Foundation (IDF) determined that 1 in 11 adults worldwide have type 2 diabetes (T2D), and 1 in 2 adults remained undiagnosed (Cho et al., 2018). The IDF further estimates that by the year 2045, the total number of diabetes cases in the world will increase from 425 million people to 629 million people (Cho et al., 2018). Postmenopausal women incur the greatest risk for type 2 diabetes (T2D) (Lindheim et al., 1994; Lynch et al., 2002). Previous studies report increased weight gain, glucose intolerance, and decreased insulin sensitivity in postmenopausal women (Carr, 2003; Pfeilschifter et al., 2002; Sites et al., 2002).

Ovariectomized (OVX) rodent models of estrogen depletion parallel postmenopausal women. OVX rodents demonstrate increased total body weight, increased, impaired glucose tolerance, insulin resistance, decreased skeletal muscle glucose uptake, and hepatic steatosis (D’Eon et al., 2005; Kumagai et al., 1993; Saengsirisuwan et al., 2009; Stubbins et al., 2012). Notably, these studies also demonstrate that estradiol (E_2_) replacement can improve these factors.

E_2_ functions by binding to two receptors, estrogen receptor (ER) α and ERβ. ERα knock‐out (KO) mice demonstrate increased adiposity (Heine et al., 2000), impaired glucose tolerance, and skeletal muscle insulin resistance (Bryzgalova et al., 2008; Riant et al., 2009), which suggests that the benefits of E_2_ may occur via ERα. In cultured adipocytes, E_2_ upregulated the insulin signaling pathway and glucose uptake, but these beneficial effects were nullified when the adipocytes were co‐treated with methylpiperidinopyrazole, a specific ERα inhibitor. Conversely, treatment with propylpyrazoletriyl (PPT), a specific ERα activator, resulted in similar improvements as seen with E_2_ (Muraki et al., 2006). Additionally, PPT treatment in obese leptin deficient ob/ob mice improved glucose tolerance and insulin sensitivity (Lundholm, Bryzgalova, et al., 2008).

Obesity alters the gut microbiome, as lean and obese human twins demonstrate significant differences in their gut microbiome (Turnbaugh et al., 2009). Furthermore, changes in gut bacteria result in changes in the body’s ability to absorb energy from food. Obese mice have an increased capacity to harvest energy from food via changes in the phyla *Bacteroidetes* and *Firmicutes* (Turnbaugh et al., 2006). The gut microbiome also regulates fat storage (Bäckhed et al., 2004).

We recently published a study showing that exercise prevents whole‐body type 2 diabetes risk factors better than E_2_ replacement in OVX rats fed a high‐fat diet, but E_2_ altered hepatic proteins involved in lipid metabolism (Fritsch et al., 2021). However, the receptors by which E_2_ provides benefits remain unknown. This study utilized the specific ERα agonist PPT and the specific ERβ agonist diarylpropionitrile (DPN) in OVX rats fed a high‐fat diet to further clarify the receptor that E_2_ works through to modulate T2D risk factors.

## 2 MATERIALS AND METHODS

### 2.1 Experimental groups

Forty‐four 186 ± 2 g female Wistar rats (Envigo) were singly housed and kept in a temperature controlled environment at 22 + 2°C on a 12 h:12 h light:dark cycle. They were fed a high‐fat diet (60% kcal fat; HFD; Research Diets D12492) for 10 weeks. Two weeks before the diet began, all of the rats underwent a bilateral ovariectomy (OVX) to remove both ovaries at 5 weeks of age. The rats were randomly assigned to one of the following five groups: (1) control (*n* = 16), (2) exercise (Ex, *n* = 8), (3) estradiol (E_2_, *n* = 8), (4) PPT (*n* = 6), and (5) DPN (*n* = 6). All of the procedures were approved by the Institutional Animal Care and Use Committee at Augustana University.

### 2.2 Exercise protocol

After 2 weeks of consuming the HFD, the Ex group was introduced to exercise on a motor‐driven treadmill for 15 min per day at 35 cm/s at a 5° incline 5 days per week. The following week, the Ex group was acclimated to the treadmill for 20 min per day at 45 cm/s at a 5° incline 5 days per week. The treatment phase (weeks 4–10) included 6 weeks of running for 25 min per day at 45 cm/s at a 5° incline 5 days per week.

### 2.3 E2, PPT, and DPN replacement

Groups received E_2_ (1.4 µg/day; Tocris Biosciences 2824), PPT (650 µg/day; Tocris Biosciences 1426), or DPN (650 µg/day; Tocris Biosciences 1494) replacement via an osmotic mini pump (ALZET model 2006) implanted subcutaneously into the upper back. Control rats received a subphysiological dose of PPT (18 µg/day) or DPN (18 µg/day). The E_2_, PPT, and DPN were dissolved in 50% sterile DMSO (Tocris 3176) and 50% sterile water according to the manufacturer’s instructions. The pumps remained in place for 6 weeks during the treatment phase of the study (weeks 4–10).

### 2.4 Food consumption

During the treatment phase (weeks 4–10), each rat, which was individually housed, was given 80–100 grams of food once per week. The following week, the amount of food left was weighed to determine to amount of food consumed during the week. The total amount of food eaten each week was divided by seven to express the data as grams eaten per day.

### 2.5 Voluntary activity cage monitoring

Each rat’s voluntary activity was continuously monitored during the dark (active) phase throughout the treatment period (weeks 4–10) using the OPTOM4 system (Columbus Instruments).

### 2.6 Dual‐energy x‐ray absorptiometry (DXA) scanning and analysis

DXA scans were performed on each rat at the beginning of week 10. Whole body rat images were obtained using a Hologic QDR 4500A/Discovery DXA densitometer with the Hologic Apex Small Animal Option. The Small Animal Step Phantom QC was performed prior to imaging. The rat was placed in the prone position and parallel to the long axis of the table. The laser was centered on the spine. The images were analyzed using Hologic Apex Software Version 5.6.0.3.

### 2.7 Intraperitoneal glucose tolerance test and insulin ELISA

Nine weeks following the start of the diet, the rats were subjected to a glucose tolerance test (GTT). The GTT occurred 72 h following exercise and after a 12 h fast. The rats were injected with glucose (2 g/kg; IP), and blood glucose levels were measured via a drop of tail blood on a glucometer (Accu‐Check Active) at 15, 30, 60, 90, and 120 min after the glucose injection. Tail blood was also collected in non‐heparinized microcapillary hematocrit tubes. The tubes were sealed with critoseal and centrifuged for 5 min in a hematocrit tube centrifuge at room temperature. The serum was collected and stored at −80°C until insulin levels were measured via an ELISA (ALPCO 80‐INSRTU). The glucose–insulin (G‐I) index was calculated using the following equation: (glucose area under the curve (AUC) X insulin AUC)/10^6.

### 2.8 Tissue harvest

Ten weeks after the start of the study, the rats were fasted for 12 h to allow us to measure chronic physiological effects of the study variables rather than acute effects of recent food consumption. Then, the rats were anesthetized with isoflurane. The anesthesia and tissue harvest occurred 72 h after the last exercise bout to allow us to measure the chronic physiological effects of exercise rather than the acute effects of exercise. The periuterine white adipose tissue (WAT) from each rat was removed and weighed. The liver was removed, frozen in liquid nitrogen, wrapped in aluminum foil, and stored at −80°C. The heart was removed to allow blood to pool in the thoracic cavity. The blood was collected into 1.5 mL tubes, allowed to clot for 30 min at room temperature, and then placed on ice. The blood was spun at 16,000 rcf, and the serum was collected and stored at −80°C.

### 2.9 Serum hormone measurements

The serum adiponectin (22‐ADPRT‐E01, ALPCO), leptin (EZRL‐83K, Millipore), ghrelin (EZRGRT‐91K, Millipore), and resistin (32–5179; ALPCO) levels were measured via an ELISA according to the manufacturer’s instructions.

### 2.10 Western blot analysis

Approximately, 50 mg of liver was homogenized in cell extraction buffer (ThermoFisher FNN0011) supplemented with 200 mM PMSF (Fisher BP231), 200 mM NaF (Sigma S6776), 200 mM sodium orthovanadate (Sigma S6508), and protease inhibitor cocktail according to the manufacturer’s instructions (Sigma P‐2714) at a ratio of 50 mg liver:600 μL buffer. The homogenized samples were rotated at 4°C for 30 min and then centrifuged at 3000 rpm for 20 min at 4°C. The supernatant was removed, and the protein concentration was determined by the Bradford Bio‐Rad Protein Assay Kit II (Bio‐Rad 5000002). The samples were mixed with 4X Bolt LDS sample buffer (ThermoFisher B0007) and 10X Bolt sample reducing agent (ThermoFisher B0009) and heated to 70°C for 10 min according to the manufacturer’s instructions. 50 ug protein were ran on 8% Bolt bis‐tris gels (ThermoFisher) according to the manufacturer’s instructions. The protein was transferred to a PVDF membrane using the Pierce Power Blotter, blocked for 1 h at room temperature in 5% milk in TBST, and then incubated in a primary antibody with gentle shaking on a rocker at 4°C overnight. The primary antibodies against acetyl‐CoA carboxylase (ACC; 3662), fatty acid synthase (FAS; 3189), and α‐tubulin (9099) were purchased from Cell Signaling Technology and used according to the manufacturer’s instructions. The primary antibody against lipoprotein lipase (LPL; 373759‐HRP), and the secondary antibody mouse IgG kappa binding protein conjugated to horseradish peroxidase (m‐IgGκ BP‐HRP; 516102) were purchased from Santa Cruz Biotechnology and used according to the manufacturer’s instructions. The secondary antibody peroxidase AffiniPure donkey anti‐rabbit IgG (H+L) (711–035–152) was purchased from Jackson Immuno Research and used according to the manufacturer’s instructions. The proteins were detected using SuperSignal West Pico PLUS chemiluminescent substrate (ThermoFisher 34577) and visualized using a UVP ChemStudio imager. The membrane was stripped with Restore Plus Western Blot Stripping Buffer (ThermoFisher 46430) for 15 min at 37°C according to the manufacturer’s instructions and re‐probed for tubulin. The protein bands were quantified using ImageJ densitometry.

### 2.11 RNA extraction, quantification, and qRT‐PCR

Total RNA was extracted from the liver using TRIzol (Invitrogen 15596026) according to the manufacturer’s instructions. The RNA was quantified using the NanoDrop Spectrophotometer and NanoDrop2000 software. The RNA was converted into cDNA using the High‐Capacity cDNA Reverse Transcription Kit (ThermoFisher 4368814) according to the manufacturer’s instructions. The cDNA was quantified using the NanoDrop Spectrophotometer and NanoDrop2000 software. qPCR was performed using the PowerUp SYBR Green Master Mix (ThermoFisher A25741) in 20 µl reactions according to the manufacturer’s instructions. All primers were purchased from Integrated DNA Technologies (IDT) and used at a concentration of 500 nM. The following primers were used: SREBP‐1c fwd 5’‐GTACCTGCGGGACAGCTTAG‐3’, SREBP‐1c rev 5’‐CAGGTCATGTTGGAAACCAC‐3’, PPARα fwd 5’‐GGCTGCTATAATTTGCTGTGG‐3’, PPARα rev 5’‐TTCTTGATGACCTGCACGAG‐3’, PPARγ fwd 5’‐GCCCTTTGGTGACTTTATGG‐3’, PPARγ rev 5’‐CATCTTCTGGAGCACCTTGG‐3’, ERα fwd 5’‐GGAAGCACAAGCGTCAGAGAGAT‐3’, ERα rev 5’‐AGACCAGACCAATCATCAGGAT‐3’, ERβ fwd 5’‐CTACAGAGAGATGGTCAAAAGTGGA‐3’, ERβ rev 5’‐GGGCAAGGAGACAGAAAGTAAGT‐3’, GAPDH fwd 5’‐TGTGGATCTGACATGCCGCC‐3’, GAPDH rev 5’‐CAGTGTAGCCCAGGATGCCC‐3’. 120 ng of cDNA was use for ERβ, and all other reactions used 10 ng cDNA. The Stratagene Mx3000P thermal cycler was used (Agilent Technologies) for cDNA amplification with the following parameters: 2 min at 50°C, 2 min at 95°C, 40 cycles of 3 s at 95°C and 30 s at 60°C, 15 s at 95°C, 1 min at 60°C, and 15 s at 95°C. All reactions were performed in triplicate, and the results from individual reactions were averaged. Changes in gene expression were determined using the 2^−ΔΔCT^ method.

### 2.12 Fecal DNA extraction, amplification, and sequencing

Fecal samples were collected at the time of sacrifice (week 10) and stored at −80°C. The DNA was isolated from 100–150 mg of sample using the QIAamp® PowerFecal® Pro DNA Kit (QIAGEN 51804). The DNA concentration was measured via a NanoDrop Spectrophotometer and then standardized to a concentration of 7.5 ng/µL. Following the 16S metagenomic sequencing library preparation, bacterial 16S ribosomal RNA gene amplicons of 390 bp were generated from the V4 region using the primers: forward 515F and reverse 926R. Amplicon PCR was performed on the DNA samples to amplify the template out of a DNA sample using region of interest specific primers with overhang adapters attached. The reactions were performed in duplicate to minimize the effects of primer preference. Following amplicon PCR, 5 µL of each sample was run through a mini agarose gel to ensure DNA amplification occurred. PCR clean‐up was performed using AMPure XP beads to purify the 16S V3 and V4 amplicon. Index PCR was then used to attach dual indices and Illumina sequencing adapters using the Nextera XT Index Kit. PCR clean‐up was performed again prior to quantification. Quantification was performed with the Qubit. The sequencing was performed using the MiSeq system (Illumina).

### 2.13 Statistical analysis

The data were statistically analyzed using IBM SPSS Statistics version 24. Normality was determined using the Shapiro–Wilk test. Normally distributed data were analyzed using a one‐way ANOVA and an LSD post‐hoc test. Non‐normally distributed data were analyzed using Kruskal–Wallis testing. The data are presented as the mean ± SE, and statistical significance was declared when *p* < 0.05.

## 3 RESULTS

### 3.1 ER activation decreases Firmicutes and increases Bacteroidetes

**FIGURE 1:**
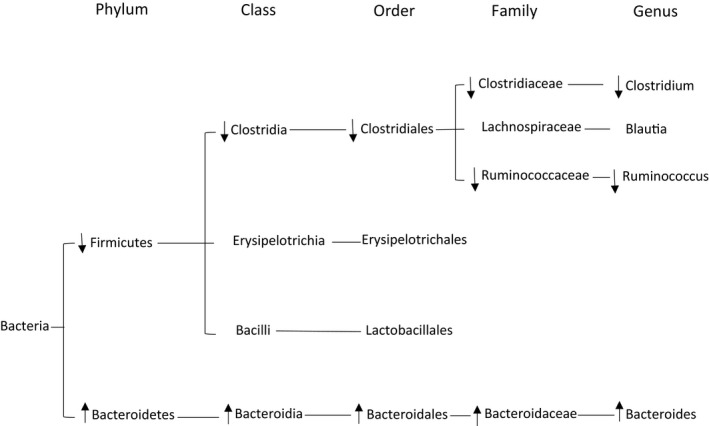
Estrogen receptor activation decreases *Firmicutes* and increases *Bacteroidetes*. After 10 weeks of high‐fat feeding in Ovariectomized female rats, fecal samples were collected. 16S rDNA sequencing was performed using the MiSeq system.

E_2_ and PPT treatment decreased the *Firmicutes* phylum, along with a subsequent decrease in the class *Clostridia*, order *Clostridiales*, family *Clostridiaceae*, and genus *Clostridium* (Table 1). While the DPN group displayed a decreased trend in these taxa, this trend was not statistically significant. Also following the decreased *Firmicutes* was a decrease in the family *Ruminococcaceae* and genus *Ruminococcus* with E_2_ and PPT treatment. While DPN treatment resulted in a non‐statistically significant decreased trend in *Ruminococcaceae*, DPN treatment resulted in a significant decrease in *Ruminococcus*. E_2_, PPT, and DPN increased the phylum *Bacteroidetes*, with a subsequent increase in the class *Bacteroidia*, order *Bacteroidales*, family *Bacteroidaceae*, and genus *Bacteroides*. A summary of changes with ER activation is shown in Figure 1. Ex decreased the order *Lactobacillales* and increased the genus *Bacteroides* (Table 1).

**TABLE 1:** Most abundant bacteria

	Abundance (%)				
	Control	Ex	E_2_	PPT	DPN
Phylum					
Firmicutes	81 ± 1.2	77 ± 1.6	71 ± 3.3*†	74 ± 1.8*	76 ± 0.7
Bacteroidetes	11 ± 0.9	15 ± 1.9	19 ± 1.9*	19 ± 3.0*	16 ± 2.1*
Proteobacteria	3 ± 0.2	3 ± 0.2	4 ± 0.3	3 ± 0.4	3 ± 0.3
Class					
Clostridia	71 ± 1.3	69 ± 1.1	57 ± 2.8*†^◊^	53 ± 4.2*†^◊^	67 ± 2.4
Bacteroidia	10 ± 0.8	13 ± 1.8	17 ± 1.6*	16 ± 3.2*	14 ± 2.1*
Bacilli	8 ± 1.1	5 ± 0.7	8 ± 2.2	7 ± 1.1	5 ± 0.4
Erysipelotrichia	2 ± 0.4	3 ± 1.6	4 ± 1.6	4 ± 1.4	5 ± 1.8
Order					
Clostridiales	70 ± 1.3	68 ± 1.2	56 ± 2.7*†^◊^	52 ± 4.3*†^◊^	60 ± 1.5
Bacteroidales	10 ± 0.8	13 ± 1.8	17 ± 1.6*	16 ± 3.2*	14 ± 2.1*
Lactobacillales	4 ± 0.5	2 ± 0.3*^	4 ± 1.1	4 ± 0.5	3 ± 1.1
Erysipelotrichales	2 ± 0.4	2 ± 0.1	3 ± 0.7	4 ± 1.4	3 ± 1.5
Family					
Lachnospiraceae	19 ± 1.2	20 ± 2.3	21 ± 1.1	15 ± 2.8	23 ± 2.3
Ruminococcaceae	22 ± 0.8	23 ± 2.0	17 ± 2.7*†	13 ± 1.3*†	18 ± 2.7
Clostridiaceae	18 ± 1.1	15 ± 2.0	10 ± 1.5*†	13 ± 1.7	17 ± 2.8
Bacteroidaceae	6 ± 0.6	9 ± 1.1	13 ± 1.1*†^	9 ± 1.3	10 ± 1.0*
Genus					
Ruminococcus	20 ± 0.8	20 ± 2.2	15 ± 2.6*^	9 ± 1.4*†	12 ± 1.3*†
Blautia	14 ± 1.3	13 ± 1.3	15 ± 1.1	10 ± 1.6	16 ± 2.4
Bacteroides	6 ± 0.6	9 ± 0.7*	13 ± 1.1*†	10 ± 2.0*	10 ± 1.7*
Clostridium	10 ± 0.7	9 ± 1.2	7 ± 1.0*†	4 ± 0.9*	5 ± 2.0

Note

Significance (*p *< 0.05): *vs. control, †vs. Ex, ^vs. PPT, ◊vs. diarylpropionitrile (DPN).

### 3.2 Body weight and abdominal WAT weight change with exercise, E_2_, and PPT treatment

**FIGURE 2:**
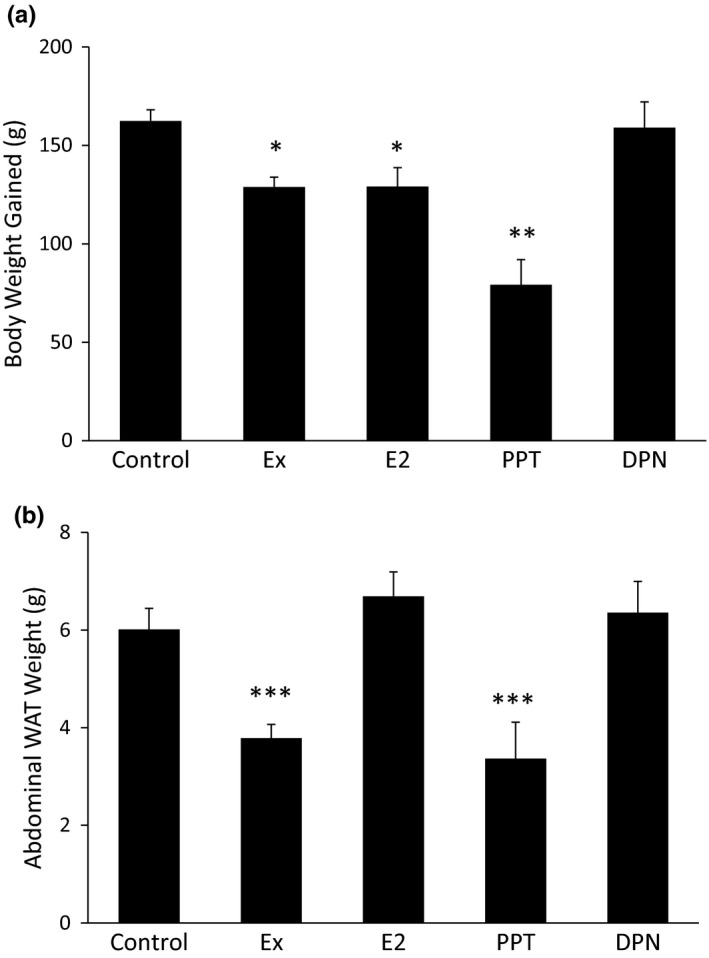
Body weight and abdominal white adipose tissue (WAT) weight changes with Ex, E_2_, and propylpyrazoletriyl treatment. Body weight was measured at baseline (week 0) and at the end of the 10 week study. The change in body weight was calculated as the difference in body weight between week 10 and week 0 (a) After week 10, the periuterine WAT was harvested and weighed (b) *p < 0.05 vs. control and diarylpropionitrile (DPN), **p < 0.001 vs. all other groups, ***p < 0.01 vs. control, E_2_, and DPN.

After 10 weeks of OVX rats consuming a HFD, the Ex and E_2_ groups gained less total body weight compared to the control and DPN groups. The PPT group gained less total body weight compared to all of the other treatment groups (Figure 2a). Although E_2_ treatment resulted in lower total body weight gained, E_2_ treatment did not decrease the abdominal WAT weight. Only the Ex and PPT treatment resulted in less abdominal WAT weight at the end of the 10 week study (Figure 2b).

### 3.3 Body composition changes with Ex, PPT, and DPN treatment

**FIGURE 3:**
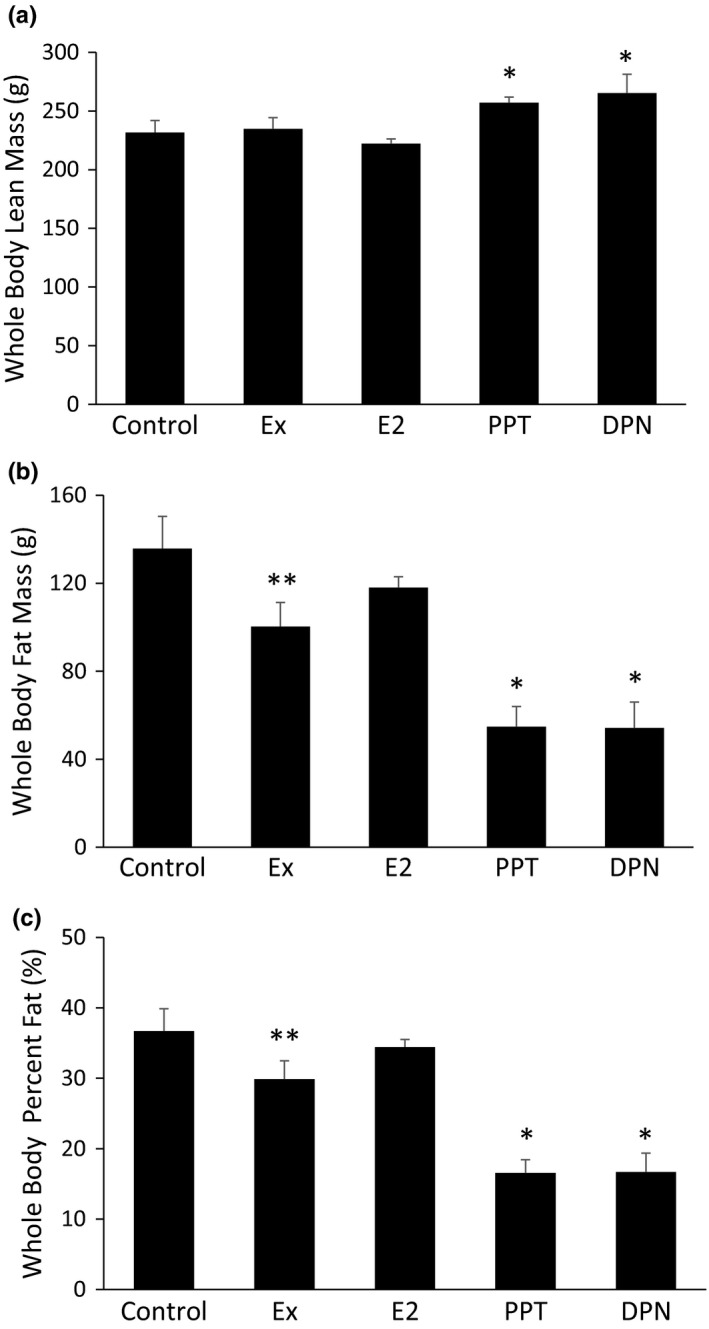
Body composition changes with Ex, propylpyrazoletriyl, and diarylpropionitrile treatment. Total body composition was measured via DXA densitometry at the beginning of week 10. The data are reported as whole body lean mass (a), whole body fat mass (b), and total percent body fat (c). *p < 0.05 vs. control, Ex, and E_2_, **p < 0.05 vs. control.

Whole body lean mass, as measured by DXA, was greater in the PPT and DPN groups (Figure 3a). Ex resulted in lower whole body fat mass (Figure 3b) and total percent body fat (Figure 3c) compared to the control. The lowest amount of whole body fat mass and total percent body fat was observed in the PPT and DPN groups, as these groups had lower whole body fat mass (Figure 3b) and lower total percent body fat (Figure 3c) compared to the control, Ex, and E_2_ groups. E_2_ treatment did not result in any changes in lean mass, fat mass, or percent body fat compared to the control.

### 3.4 PPT decreases food consumption and E_2_, PPT, and DPN increase voluntary cage movement

**FIGURE 4:**
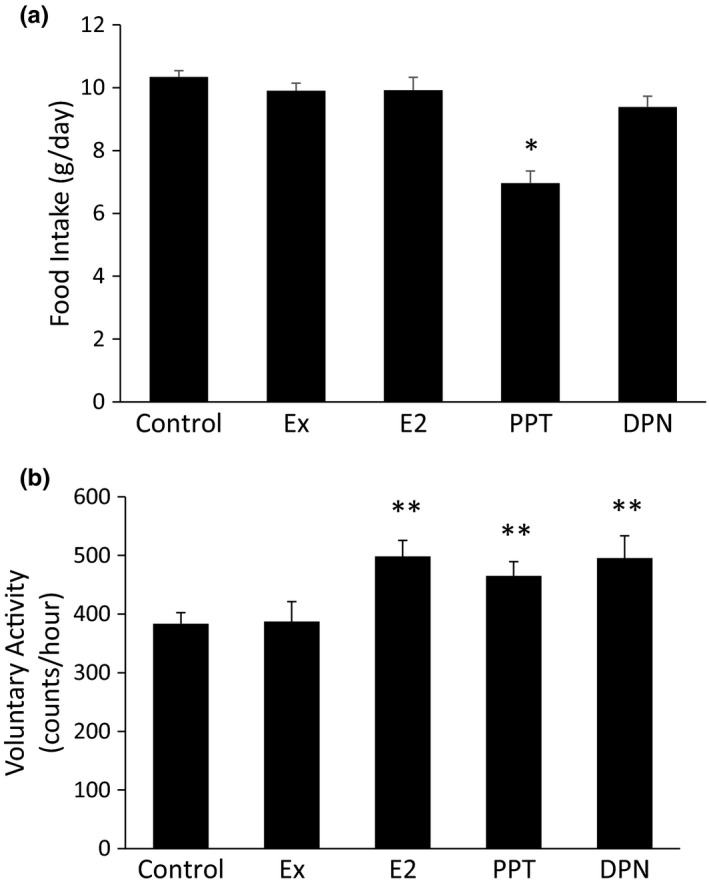
Propylpyrazoletriyl (PPT) decreases food consumption and E_2_, PPT, and diarylpropionitrile increase voluntary cage movement. Food consumption was measured throughout the treatment phase (weeks 4–10) (a). Voluntary cage activity was continuously monitored during the dark (active) phase throughout the treatment phase (b). *p < 0.05 vs. all other groups, **p < 0.05 vs. control and Ex.

Caloric intake via food consumption and caloric expenditure via movement are two contributing factors to total body weight and body fat mass. Only the PPT group demonstrated reduced food consumption compared to the control group, with no change in food intake with Ex, E_2_, or DPN (Figure 4). Voluntary cage activity was increased in the E_2_, PPT, and DPN groups, with no change in voluntary cage activity in the Ex group compared to the control (Figure 4b).

### 3.5 Exercise improves blood glucose and serum insulin levels during a GTT

**FIGURE 5:**
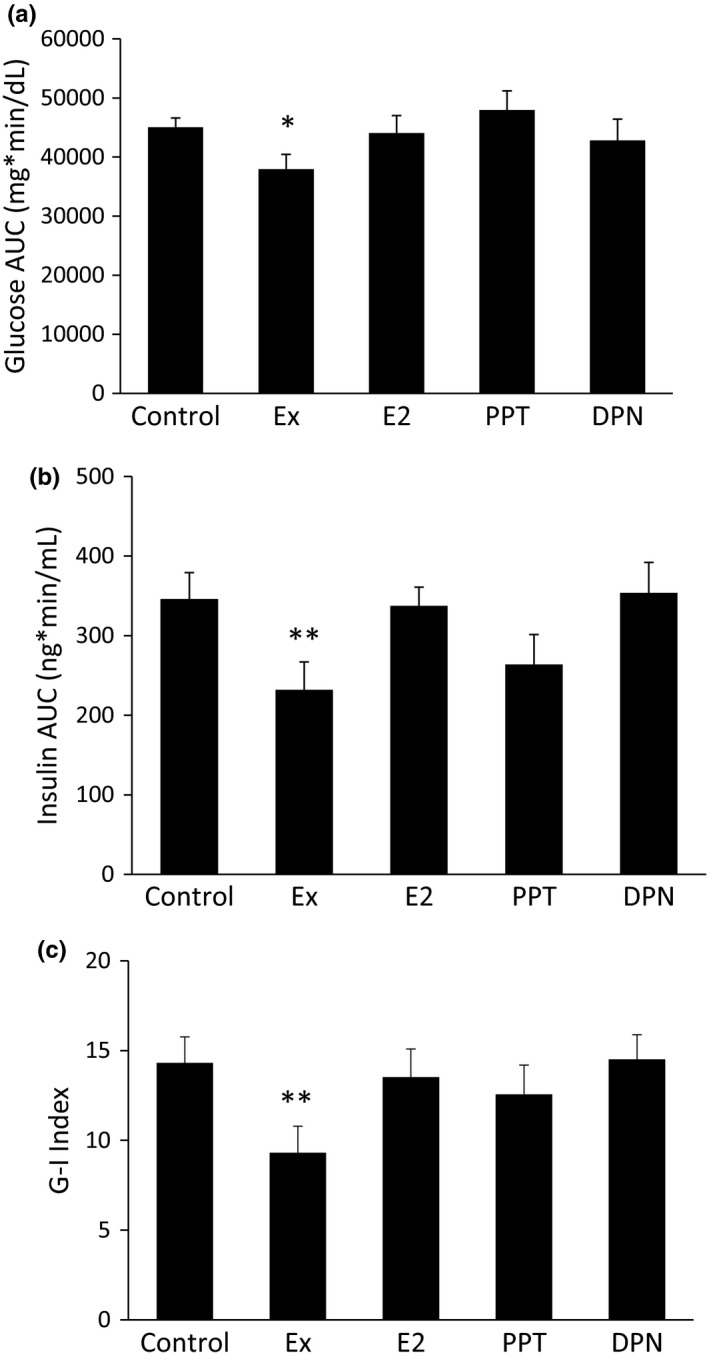
Exercise improves blood glucose and serum insulin levels during a glucose tolerance test (GTT). After 9 weeks, the rats were fasted for 12 h prior to an intraperitoneal GTT. Blood glucose measurements were taken during the GTT, and tail blood was collected at each time point to measure the serum insulin levels via an ELISA. The area under the curve (AUC) for the blood glucose levels (a) and plasma insulin levels (b) throughout the GTT was calculated. The G‐I Index was calculated from the glucose and insulin AUC (c). *p < 0.05 vs. control and propylpyrazoletriyl, **p < 0.05 vs. control, E_2_, and diarylpropionitrile.

Total body weight and abdominal adipose tissue are often predictive factors for blood glucose and insulin regulation. While this study shows that E_2_ and PPT decreased total body weight, abdominal WAT weight, and/or total body percent fat, only Ex decreased blood glucose (Figure 5a) and serum insulin (Figure 5b) levels during the GTT. The G‐I Index was also lower in the Ex group (Figure 5c). E_2_, PPT, and DPN treatments did not lower the blood glucose or serum insulin levels.

### 3.6 Serum hormone changes with Ex, E_2_, PPT, and DPN treatment

**FIGURE 6:**
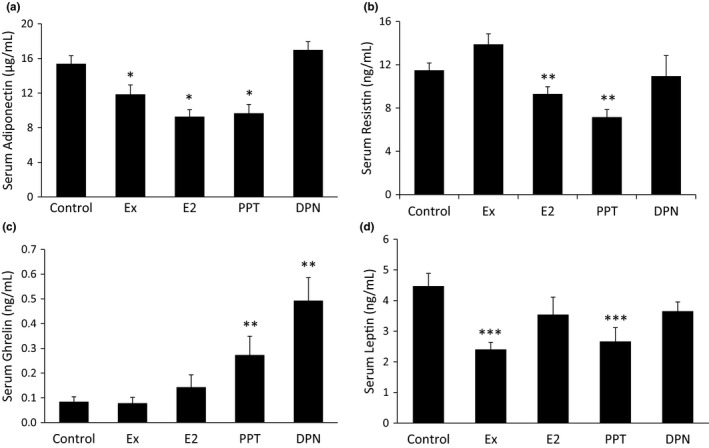
Serum hormone changes with Ex, E_2_, propylpyrazoletriyl, and diarylpropionitrile (DPN) treatment. At the end of the 10 week study, blood was collected to measure the serum adiponectin (a), resistin (b) ghrelin (c), and leptin (d) levels via an ELISA. *p < 0.05 vs. control and DPN, **p < 0.05 vs. control and Ex, ***p < 0.05 vs. control.

Adiponectin and resistin can influence glucose regulation, and ghrelin and leptin can influence food intake. Ex, E_2_, and PPT decreased serum adiponectin compared to the control, and DPN treatment had no effect (Figure 6a). Only E_2_ and PPT treatment decrease serum resistin levels, with no change in resistin due to Ex or DPN (Figure 6b). Serum ghrelin levels increased with PPT and DPN treatment (Figure 6c), and serum leptin decreased in the Ex and PPT groups (Figure 6d).

### 3.7 Hepatic proteins and transcriptional regulators of fat metabolism

**FIGURE 7:**
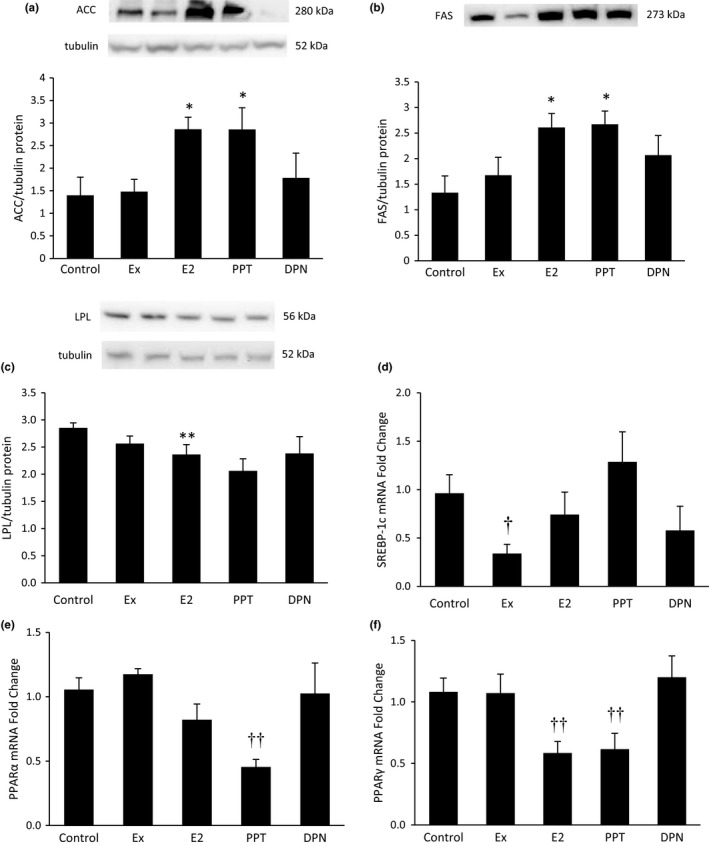
Hepatic proteins and transcriptional regulators of fat metabolism. After 10 weeks of high‐fat feeding in Ovariectomized female rats, the liver was harvested from anesthetized rats and frozen in liquid nitrogen. Western blot analyses measured proteins involved in fat regulation including ACC (a), FAS (b), and LPL (c). Panels A and B used the same loading control (tubulin). qRT‐PCR was used to assess mRNA levels of transcriptional regulators of fat metabolism including SREBP‐1c (d), PPARα (e), and PPARγ (f). *p < 0.05 vs. control and Ex, **p < 0.05 vs. control, ^†^ *p *< 0.05 vs. control and propylpyrazoletriyl, ^††^ vs. control, Ex, and diarylpropionitrile.

The proteins ACC and FAS are involved in de novo lipid synthesis. ACC (Figure 7a) and FAS (Figure 7b) were upregulated with E_2_ and PPT treatment. Conversely, LPL, a protein that stimulates lipid uptake, decreased with E_2_ and PPT treatment (Figure 7c). SREBP‐1c, PPARα, and PPARγ are transcriptional regulators of proteins involved in fat metabolism. While, Ex decreased SREBP‐1c mRNA expression (Figure 7d), PPT decreased PPARα mRNA expression (Figure 7e). Both E_2_ and PPT treatment decreased PPARγ mRNA expression (Figure 7f).

### 3.8 Hepatic mRNA ERα and ERβ expression

**FIGURE 8:**
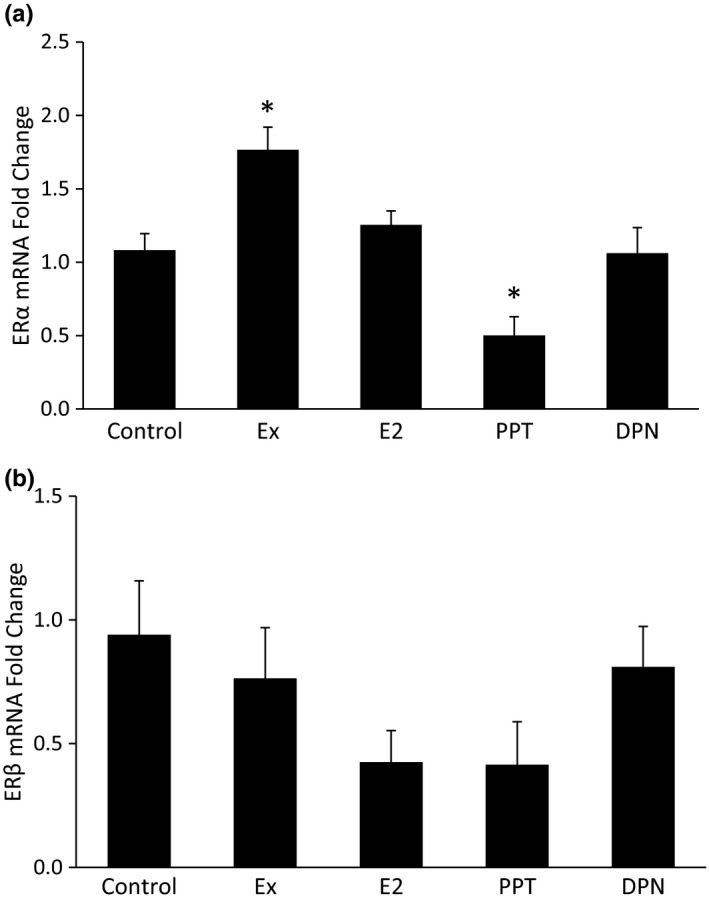
Hepatic mRNA estrogen receptor (ER)α and ERβ expression. After 10 weeks of high‐fat feeding in Ovariectomized female rats, the liver was harvested from anesthetized rats and frozen in liquid nitrogen. qRT‐PCR was used to assess mRNA levels of ERα (a) and ERβ (b) in the liver. *p < 0.05 vs. all other groups.

The physiological effects of E_2_, PPT, and DPN are carried out via ERα and ERβ. Receptor expression levels are important factors to consider when treating with hormones and/or drug agonists. Ex increased hepatic ERα mRNA expression, and PPT decreased hepatic ERα mRNA expression (Figure 8a). Treatment with Ex, E_2_, PPT, or DPN did not result in statistically significant changes in hepatic ERβ mRNA expression (Figure 8b).

## 4 DISCUSSION

This study assess the changes in several T2D risk factors due to exercise and E_2_. The study further explores the E_2_ paradigm by using specific ERα and ERβ agonists. E_2_, PPT, and/or DPN modulated all of the outcomes measured in this study except for blood glucose and serum insulin. Only Ex effectively decreased blood glucose and serum insulin levels.

Obesity and E_2_ replacement can alter the gut microbiome. The phylum *Bacteroidetes* is decreased in obese humans, and is restored with weight loss and a low‐calorie diet (Ley et al., 2006). In obese leptin deficient ob/ob mice, *Bacteroidetes* is also decreased, and an increase in the phylum *Firmicutes* also occurs (Ley et al., 2005). Other studies show that E_2_ replacement following OVX in rodents positively influences the gut microbiome. E_2_ replacement decreased *Firmicutes* and increased *Bacteroidetes* (Acharya et al., 2019), which is in accordance with our data. However, the Acharya et al. study did not examine specific ER activation. Our study adds novel information to the literature, showing that ERα activation, but not ERβ, decreases the *Firmicutes* phylum. We further show that both ERα and ERβ activation are responsible for the increased *Bacteroidetes* phylum. As *Firmicutes* increases and *Bacteroidetes* decreases with obesity (Ley et al., 2005, 2006), the opposing action via ER activation we show in our present study (i.e., decreased *Firmicutes* and increased *Bacteroidetes*) may provide beneficial effects via restoring the gut microbiome. Our data provide a broad overview of the changes in the gut microbiome with ERα and ERβ activation.

Male and female humans and rodents have differing native gut species (Jašarević et al., 2016; Org et al., 2016). While a possible link between metabolic factors and reproductive hormones remains elusive, one important hormone to consider is leptin. The presences of estrogens can alter neurogenesis in the brain, including leptin sensitive regions (Bless et al., 2014). E_2_ also increases leptin sensitivity in female and male rodents (Clegg et al., 2003). Given leptin’s role in promoting satiety and regulating fat storage and metabolism, the interaction between E_2_ and leptin to regulate the gut microbiome deserves further attention.

Caloric intake and caloric expenditure drastically influence body weight and body composition, which are significant risk factors for T2D. In our study, Ex decreased body weight, abdominal WAT, and whole body fat mass without increasing lean mass, which one would expect with aerobic training. PPT and DPN increased lean mass while decreasing fat mass. Selective ERβ activation has been previously shown to increase lean mass and decrease fat mass (Yepuru et al., 2010). Additionally, E_2_ is a known anabolic hormone that contributes to increased lean mass, although testosterone results in much greater gains in muscle mass (Enns & Tiidus, 2010; Sipilä et al., 2001). The PPT group also had the lowest body weight gain and reduced WAT, which could be contributed to the decreased food intake and increased voluntary cage activity. Specific ERα activation in the brain suppresses food intake (Zhu et al., 2015). Additionally, the presence of E_2_ increases voluntary activity (Duval et al., 2013; Izumo et al., 2012; Rogers et al., 2009). Our current study supports this and further adds to the literature that E_2_ provides this function through both ERα and ERβ.

Although activation of ERα, and to some extent ERβ, modulated several outcomes measured in this study, ER activation via E_2_, PPT, or DPN did not alter blood glucose or serum insulin levels. However, we did show that Ex decreased the glucose and insulin AUC during a glucose tolerance test, which signifies improved glucose control and improved insulin sensitivity. The beneficial effect of Ex decreasing blood glucose and serum insulin levels without improvements due to E_2_ has been shown previously (Fritsch et al., 2021). In that study, Ex treatment alone or in combination with E_2_ replacement decreased blood glucose and serum insulin in OVX rats fed a HFD, but E_2_ treatment alone did not show similar improvements. E_2_ treatment alone did alter body weight gain and hepatic ACC, pACC, FAS, and LPL protein levels. With several metabolic changes occurring due to E_2_ treatment, the lack of improvement in glycemic control is somewhat surprising.

Adipokines such as adiponectin, resistin, and leptin play a role in insulin sensitivity and food intake. Specifically, adiponectin and resistin may play a role in insulin resistance. Adiponectin may reduce insulin resistance and promote insulin sensitization (Yamauchi et al., 2002). However, adiponectin is often inversely associated with obesity, with increased adiposity associated with decreased adiponectin (Arita et al., 1999), and hence, increased insulin resistance. Several studies demonstrate that the loss of estrogens resulted in increased serum adiponectin (D’Eon et al., 2005; Gavrila et al., 2003; Leung et al., 2009; Ludgero‐Correia et al., 2012). Accordingly, our study shows that E_2_ replacement decreased serum adiponectin, which has also been shown in OVX mice (Bryzgalova et al., 2008; Riant et al., 2009). Notably, we add novel information to the literature by showing that these effects of E_2_ are mediated via ERα, as the PPT group also demonstrated decreased adiponectin while the DPN group did not. Resistin is synthesized and secreted from the adipose tissue and may contribute to insulin resistance. In our study, E_2_ treatment decreased serum resistin levels, which agrees with a previous report that treated OVX mice with E_2_ for 60 days (D’Eon et al., 2005). In our study, the PPT group also demonstrated decreased resistin, suggesting that E_2_ decreases serum resistin via ERα activation. Our results differ from Yuan et al. showing E_2_ treatment for 20 days in OVX mice fed a HFD did not alter resistin levels (Yuan et al., 2015). Our study gave E_2_ and PPT replacement for 42 days. Therefore, the length of hormone replacement may be an important factor modulating serum resistin. Additionally, OVX in mice increases resistin mRNA in the adipose tissue (Gui et al., 2004), and E_2_ replacement in OVX rats decreases resistin mRNA in the adipose tissue (D’Eon et al., 2005; Huang et al., 2005), which is in accordance with our data. Taken together, the presence of E_2_, either endogenous or replacement, working through ERα may preserve insulin sensitivity via decreasing resistin.

Ghrelin and leptin are important hormones of hunger and satiety. Ghrelin is secreted from an empty stomach and stimulates feelings of hunger. Our data show that both PPT and DPN increased serum ghrelin, yet E_2_ treatment did not significantly alter ghrelin levels. A previous study notes that the gastric ghrelin protein level was decreased with E_2_ treatment in OVX rats, but the plasma ghrelin levels remained unchanged (Yokota‐Nakagi et al., 2020). More research is needed to clarify the impact of ER activation on ghrelin’s actions. Leptin is secreted from the WAT and simulates feelings of satiety. We and others have previously shown that exercise decreases serum leptin levels (Essig et al., 2000; Fritsch et al., 2021; Jacobs et al., 2020; Metz et al., 2016). The effect of E_2_ on serum leptin levels remains unknown. While some studies show that E_2_ treatment decreases serum leptin in rats (D’Eon et al., 2005; Weigt et al., 2012), another study shows that hormone replacement therapy in human females does not decrease serum leptin (Kohrt et al., 1996). Whether E_2_ alters leptin levels may depend on the extent to which the ERs are activated, as our present study shows that PPT treatment decreased leptin levels and DPN did not. While selectively activating one or the other ER does not occur in nature, the expression of the ERs could change, which would allow E_2_ to preferentially activate one receptor over the other.

Transcriptions factors such as SREBP‐1c, PPARα, and PPARγ regulate genes involved in fat metabolism. Our study shows that Ex decreased hepatic SREBP‐1c mRNA, which agrees with Pighon et al. but disagrees with Zheng et al. showing increased hepatic SREBP‐1c mRNA with treadmill training (Zheng et al., 2018). Decreased SREBP‐1c expression with exercise may reduce hepatic steatosis and T2D risk factors because SREBP‐1c regulates several genes involved in fat metabolism. Two genes regulated by SREBP‐1c that we measured include FAS and ACC (Griffin & Sul, 2004), which are involved in de novo lipid synthesis. Although Ex modulated SREBP‐1c in our study, FAS and ACC protein expression did not change with Ex, which is in accordance with our previous research (Fritsch et al., 2021). Additionally, we previously reported that E_2_ treatment increases hepatic FAS and ACC protein expression in OVX rats fed a HFD (Fritsch et al., 2021). The present study also shows the same increase and further adds to the literature that this increase occurs via ERα activation.

PPARα promotes fatty acid oxidation by upregulating proteins involved in fatty acid oxidation, and PPARγ can increase the expression of genes involved in increased fat uptake and lipogenesis (Vacca et al., 2011). Some studies show that hepatic PPARα mRNA expression doesn’t change with OVX or OVX+E_2_ replacement (D’Eon et al., 2005; Weigt et al., 2013; Yoon et al., 2015; Zoth et al., 2012), which agrees with our study. Yet another study shows that E_2_ replacement in OVX rats fed a HFD decreases the hepatic PPARα protein expression (Buniam et al., 2019). As specific ERα activation via PPT resulted in decreased PPARα expression in our study, our study adds novel information to the literature by showing that the extent to with ERα and ERβ are activated may be important factors. In contrast, other studies show that hepatic PPARα mRNA increased in OVX rodents with E_2_ replacement (Paquette et al., 2008; Pighon et al., 2011). However, these rodents were fed a standard diet, and the rats in our study were fed a HFD. Therefore, diet and the ratio of ERα and ERβ activation may be important factors to consider when studying PPARα regulation. Our current study and others (Buniam et al., 2019; Lundholm, Zang, et al., 2008; Weigt et al., 2013) clearly show that E_2_ treatment decreases PPARγ expression. Our current study further adds to the literature by showing that E_2_ exerts its effects via ERα activation. E_2_, via ERα, may provide protection against T2D risk factors by decreasing the expression of genes involved in fat uptake and lipogenesis.

One gene regulated by PPARγ is LPL, which promotes fat uptake (Wong & Schotz, 2002). Our study shows that both E_2_ and PPT treatment decreased hepatic PPARγ, and hepatic LPL protein was also decreased in the E_2_ and PPT groups. We previously reported that hepatic LPL protein decreased with E_2_ treatment in OVX rats fed a HFD (Fritsch et al., 2021). The present study also shows the same decrease and further adds novel information to the literature that this decrease is via ERα activation, which has not been previously shown.

In our current study, we see that total body weight gained, abdominal WAT weight, food intake, hormones regulating insulin sensitivity and food intake, hepatic proteins and transcription factors involved in fat regulation, and the gut microbiota are differentially regulated via ERα and ERβ activation. Studies that only assess the effects of E_2_ on these variables miss important pieces of information. Our study provides crucial data on whether ERα and/or ERβ activation can modify various risk factors for T2D. While selective ER activation doesn’t necessarily occur in nature, ER expression can change. Our study demonstrates increased hepatic ERα expression with Ex. In this scenario, any endogenous or exogenous E_2_ could preferentially activate ERα over ERβ and result in the physiological changes associated with ERα activation. More studies need to examine individual ER activation and their physiological effects, along with studying the changes in ER expression.

While numerous studies assess the effects of E_2_ replacement in OVX rats fed a standard diet (D’Eon et al., 2005; Kumagai et al., 1993; Saengsirisuwan et al., 2009), fewer studies include high‐fat feeding/obesity as a study variable. As obesity rates in the United States and throughout the world are increasing, studying E_2_ replacement in the context of obesity remains vital. Currently, E_2_ replacement is not a recommended treatment for T2D (Grossman et al., 2017). While HRT in postmenopausal humans can decrease the risk of T2D (Espeland et al., 1998; Gower et al., 2006; Kanaya et al., 2003; Margolis et al., 2004), HRT also increases the risk of thrombosis, breast cancer, stroke, and coronary artery disease (Nelson et al., 2002). Notably, exercise can reduce the risk of T2D and its associated factors such as hyperglycemia, hyperinsulinemia, and hepatic steatosis (Goedecke & Micklesfield, 2014). Therefore, studying exercise as a treatment remains essential.

## AUTHOR CONTRIBUTIONS

The experiments were performed at Augustana University. BKG contributed to the conception and design of the work, acquisition, analysis and interpretation of data for the work and drafting the work and revising it critically for important intellectual content. JAS and KAG contributed to acquisition, analysis and interpretation of data for the work and drafting the work and revising it critically for important intellectual content. All authors approved the final version of the manuscript and agree to be accountable for all aspects of the work in ensuring that questions related to the accuracy or integrity of any part of the work are appropriately investigated and resolved. All persons designated as authors qualify for authorship, and all those who qualify for authorship are listed.

## CONFLICT OF INTERESTS

None of the authors of this paper have a competing interest.
